# Rethinking Left Ventricular Noncompaction and Excessive Left Ventricular Trabeculation: Development, Genetics, and a Proposed Conceptual Framework

**DOI:** 10.3390/jcm15187175

**Published:** 2026-09-15

**Authors:** Keiichi Hirono

**Affiliations:** Department of Pediatrics, Toyama University Hospital, Toyama 930-0194, Japan; khirono1973@gmail.com; Tel.: +81-76-434-7313

**Keywords:** left ventricular noncompaction, excessive left ventricular trabeculation, ventricular development, genetics, cardiomyopathy, phenotype

## Abstract

Left ventricular noncompaction (LVNC) has long been treated as a distinct cardiomyopathy defined by excessive left ventricular trabeculation. Human developmental studies no longer support the classical arrest-of-compaction hypothesis and instead favor differential growth of the trabecular and compact layers. Excessive trabeculation is also observed in inherited and developmental cardiomyopathies, as a change accompanying other cardiac disease, in asymptomatic individuals, and reversibly in pregnancy and in athletes. Morphology alone is therefore insufficient to define a disease entity. This narrative, conceptual review re-examines ventricular wall formation, the mechanisms producing excessive trabeculation, genetic heterogeneity, and clinical phenotypes and shows that no single axis organizes LVNC unequivocally. Integrating these elements, excessive trabeculation is organized into three clinical pathophysiologic contexts: primary cardiomyopathy with hypertrabeculation, in which overt cardiomyopathy is not sufficiently explained by another underlying disease or by pathological hemodynamic loading; disease-associated hypertrabeculation; and hypertrabeculation without overt cardiomyopathy. The purpose of this organization is to isolate a candidate disease entity that can be tested, using the attribution step by which current definitions of hypertrophic and dilated cardiomyopathy are themselves constructed. The framework is built on three axes: category, descriptive phenotypic group, and risk modifiers. It is hypothesis-generating; operationalization, interobserver reproducibility, prospective outcomes and external validation remain to be realized.

## 1. Introduction: Problems Inherent in the Disease Concept of LVNC

Excessive left ventricular trabeculation (left ventricular hypertrabeculation), which has been described as left ventricular noncompaction (LVNC), is a morphological phenotype characterized by prominent trabeculae and deep intertrabecular recesses. Since the initial report by Chin et al. in 1990, its clinical presentation has been recognized as remarkably diverse, ranging from an asymptomatic state to severe heart failure, lethal arrhythmias, and thromboembolism [1]. Historically, it has been treated as a distinct form of inherited cardiomyopathy [2]. More recently, however, both the developmental premise underlying the term “noncompaction” itself and the practice of translating a morphological finding directly into a disease diagnosis have been reconsidered [3,4,5].

The findings that prompted this reconsideration can be broadly divided into three groupings. First, in the human ventricular wall there is little morphometric evidence that the trabecular layer disappears or coalesces and is converted into the compact layer; rather, a developmental model in which wall architecture changes through differential growth of the trabecular and compact layers is supported [6,7,8]. Second, excessive trabeculation also appears in conditions such as pregnancy and high-intensity exercise and may regress after the load is relieved, so morphology alone cannot be regarded as evidence of a pathological cardiomyopathy [9,10]. Third, the genetic background associated with LVNC spans developmental, sarcomeric, ion channel, and metabolic and mitochondrial genes, with substantial genetic overlap with other cardiomyopathies, and the diagnostic yield of genetic testing remains approximately 30–45%, varying with the cohort, the panel used and the criteria for variant classification [11,12,13,14]. Moreover, large-scale analyses using cardiac magnetic resonance imaging in the general population indicate that myocardial trabeculation is not a binary disease morphology but a continuous quantitative trait influenced by both common and rare variants [15].

Terminology and position taken in this review: Because the developmental premise that trabeculae coalesce into the compact layer is not supported by current evidence [6,7], a term carrying that premise is avoided as the name of a disease entity. This review does not regard LVNC morphology itself as an independent disease entity; a subset of cases with this morphology nevertheless behaves as a primary cardiomyopathy, and this subset is designated Category 1 (primary cardiomyopathy with hypertrabeculation) in Section 7. The terms used throughout, and the conventions governing them, are set out in Box 1.

Box 1 **Terminology used in this review.**

Excessive trabeculation (equivalently, hypertrabeculation) denotes the morphological finding. Left ventricular noncompaction (LVNC) is retained only when referring to pre-viously published cohorts, criteria or guidelines that used that term. Primary cardio-myopathy with hypertrabeculation denotes the clinical pathophysiologic context (Cat-egory 1) and has previously been termed noncompaction cardiomyopathy; it is not as-serted here as an established disease entity (Section 7.1). The hyphenated form “non-compaction” is not used. P/LP variant denotes a variant classified as pathogenic or likely pathogenic.

The aim of this review is to re-examine, in a continuous manner, normal ventricular wall formation, pathogenetic mechanisms, genetic background, and clinical phenotypes and, thereby, to organize the heterogeneity of the conditions that have been grouped together as LVNC. When the knowledge from each of these layers is integrated in turn, it becomes apparent that the conventional approach of using morphology, genotype, or clinical phenotype alone as the organizing axis is insufficient and that it is necessary to distinguish the clinical pathophysiologic context in which excessive trabeculation appears. As the integrated consequence of this analysis, three clinical pathophysiologic contexts are proposed, and their clinical implications and limitations are discussed. Throughout, this organization is presented as a proposed conceptual framework that requires prospective clinical validation.

### Literature Search

This is a narrative, conceptual review and was not conducted as a systematic review. PubMed/MEDLINE and Embase were searched from inception to [August 2026] (last search [1]) using combinations of “left ventricular noncompaction”, “excessive trabeculation”, “hypertrabeculation”, “ventricular wall development”, “gene–disease validity” and “cardiomyopathy”, without language restriction; the reference lists of retrieved articles were screened manually. Articles were selected for relevance to ventricular wall development, gene–disease validity, or clinical outcome; no formal quality scoring was applied, and where reports conflicted, both are cited. Guideline statements were verified against the primary guideline documents, and gene–disease validity against the ClinGen gene curation records (accessed [1]) together with the published ClinGen-based systematic assessment [14]. Several of the clinical statements rest on heterogeneous retrospective pediatric cohorts, including studies from the author’s own group; these are identified where cited, and no conclusion of this review rests on them alone. This constraint is restated in Section 7.6.

## 2. Formation of the Normal Ventricular Wall

### 2.1. Trabeculation and Growth of the Compact Layer

In early embryonic development, the ventricular wall is largely composed of trabeculae, myocardial projections lined by the endocardium, which develop radially toward the ventricular cavity. Trabeculae are thought to increase the myocardial surface area and to enhance the efficiency of gas exchange and nutrient diffusion at a stage when coronary circulation is not yet developed. In the mouse, trabeculation begins around embryonic day nine, when cardiomyocytes protrude toward the ventricular lumen, after which the trabecular layer and the epicardial-side compact layer each grow with their own proliferative dynamics [16,17].

Growth of the ventricular wall is not undirected cell proliferation but is oriented. In the postnatal mouse ventricular wall, an endocardial-side *Nppa*-positive layer, an epicardial-side *Hey2*-positive layer, and an intermediate region in which both are intermingled (hybrid zone) have been identified, and inhibition of the expansion of *Hey2*-positive cardiomyocytes during embryonic life results in persistent excessive trabeculation after birth [18]. Thus, the trabecular and compact layers possess independent proliferation and differentiation programs, and the quantitative balance between them determines ventricular wall morphology. It should be noted, however, that detailed morphological descriptions of the onset of trabeculation in the human fetal heart are scarce and that much of the cellular-level mechanistic knowledge derives from the mouse and zebrafish.

### 2.2. Re-Examination of the Concept of “Compaction”: From Coalescence to Differential Growth

In the conventional model, trabeculae were held to coalesce from the epicardial side toward the endocardial side in late embryonic development to form the compact layer, and arrest of this “compaction” was held to leave the trabeculated, fetal-type myocardium in place [1]. Subsequent systematic morphometry of the human ventricular wall, however, does not support this premise.

Faber et al. comprehensively examined quantitative data on the ventricular wall across human, mouse, and chicken developmental series and showed that a decrease in the absolute amount of the trabecular layer, which should be an obligatory consequence of compaction, has not been documented in humans [6]. Both the trabecular and the compact layers continue to grow, and the ratio between them declines over time because the latter grows faster. A marked increase in the compact layer with a reciprocal decrease in the trabecular layer is clearly observed only in the chicken, indicating substantial interspecies differences. Gestational-age-specific morphometry in the mouse and human has likewise shown that changes in ventricular wall morphology can be described as a difference in growth rate between the two layers (differential growth) [8]. The same group further demonstrated that, in the human heart, trabecular and compact-wall cardiomyocytes have an equivalent capacity for force generation, with no essential difference in the amounts of contractile apparatus or mitochondria-related proteins [7]. This finding is also inconsistent with the interpretation of trabeculae in adult LVNC as the “persistence of immature, fetal-type myocardium” [19].

**Figure 1:**
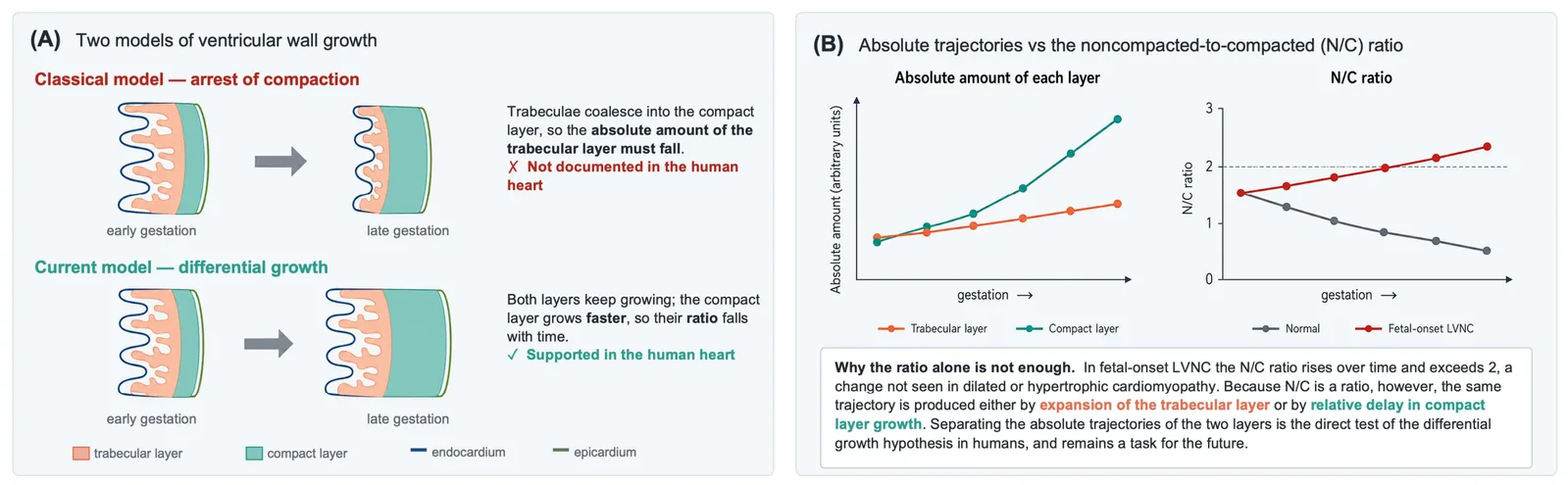
Formation of the ventricular wall: from arrest of compaction to differential growth. (**A**) Two models of ventricular wall growth. In the classical model, trabeculae coalesce from the epicardial toward the endocardial side during late gestation to form the compact layer, so that the absolute amount of the trabecular layer must fall. Systematic morphometry of the human heart has not documented such a decrease [6]. In the current model, both the trabecular and the compact layer continue to grow, and the compact layer grows faster, so that the ratio between them declines with time; this differential growth describes the human and mouse ventricular wall [6,8]. Trabecular and compact-wall cardiomyocytes generate equivalent force and show no essential difference in contractile or mitochondria-related protein content, which is inconsistent with the interpretation of trabeculae as a persistent fetal-type myocardium [7,19]. The drawings are schematic and are not to scale. (**B**) Absolute trajectories of the two layers and the corresponding noncompacted-to-compacted (N/C) ratio. In fetal-onset LVNC, the N/C ratio increases over time and exceeds two throughout the perinatal period, a change not observed in dilated or hypertrophic cardiomyopathy [21,22]. Because N/C is a ratio, an identical trajectory is produced either by expansion of the trabecular layer or by relative delay in growth of the compact layer; evaluating the absolute trajectories of the two layers separately is the direct test of the differential growth hypothesis in humans and remains a task for the future. The curves illustrate the relationships described in the cited studies and do not reproduce measured values; the vertical axes are in arbitrary units. Unless otherwise indicated, the mechanisms depicted derive from animal models and in vitro systems; the human evidence is confined to the morphometric observations shown.

Accordingly, the process is now more appropriately understood not as the “arrest of compaction” but as a “deviation in the growth balance between the trabecular and compact layers” (Figure 1A) [3,6,7,8,19]. However, since this conclusion is most strongly supported in humans, whereas in the mouse the existence of a limited coalescence process cannot be entirely excluded and in the chicken coalescence is prominent, oversimplification should be avoided.

Longitudinal observations of fetal-onset cases in Japan reinforce this framework from the side of clinical data. In fetal-onset LVNC, the ratio of the thickness of the noncompacted to the compacted layer (noncompacted-to-compacted ratio; N/C ratio) increases over time and exceeds two—the end-systolic echocardiographic threshold of Jenni et al. [20]—throughout the perinatal period, whereas comparable changes are not observed in dilated or hypertrophic cardiomyopathy [21,22]. This finding suggests that formation of the trabecular layer continues into late gestation. Because the N/C ratio is a ratio, however, it cannot discriminate whether the increase reflects expansion of the trabecular layer or relative delay in growth of the compact layer (Figure 1B). Evaluating the absolute trajectories of the two layers separately is a task for the future and would directly test the differential growth hypothesis in humans. In addition, large-scale cardiac magnetic resonance (CMR) morphological analyses in the general population have shown that the degree of trabeculation is a continuous quantitative trait influenced by both common and rare variants [15], supporting the limitations of treating LVNC morphology as binary.

### 2.3. Molecular Identity of Trabecular and Compact Myocardium

Single-cell RNA sequencing and spatial transcriptomics have demonstrated at the molecular level that trabecular and compact myocardia have distinct transcriptional profiles. Spatial analysis of the human fetal heart has revealed that ventricular cardiomyocytes are organized in layers as multiple subpopulations spanning the wall, forming spatially organized cellular communities [23]. In the mouse, the trabecular layer expresses *Nppa*, Bmp10, and *Irx3* and the compact layer expresses *Hey2*, with an intermediate population co-expressing markers of both groups at their boundary [18,24].

These findings indicate that, at least in experimental models, excessive trabeculation cannot be explained solely as “a state in which trabeculae failed to be physically compacted” and may also arise from abnormalities in the specification, maintenance, or spatial arrangement of compact-layer cardiomyocytes. In *Prdm16* deficiency, compact-layer cardiomyocytes shift toward a trabecular-like transcriptional identity [24]. However, an equivalent identity conversion has not been directly demonstrated in the human LVNC heart, and verification by longitudinal single-cell and spatial analyses of the human fetal heart is required.

### 2.4. Coordination with Coronary Vascular Formation

**Figure 2:**
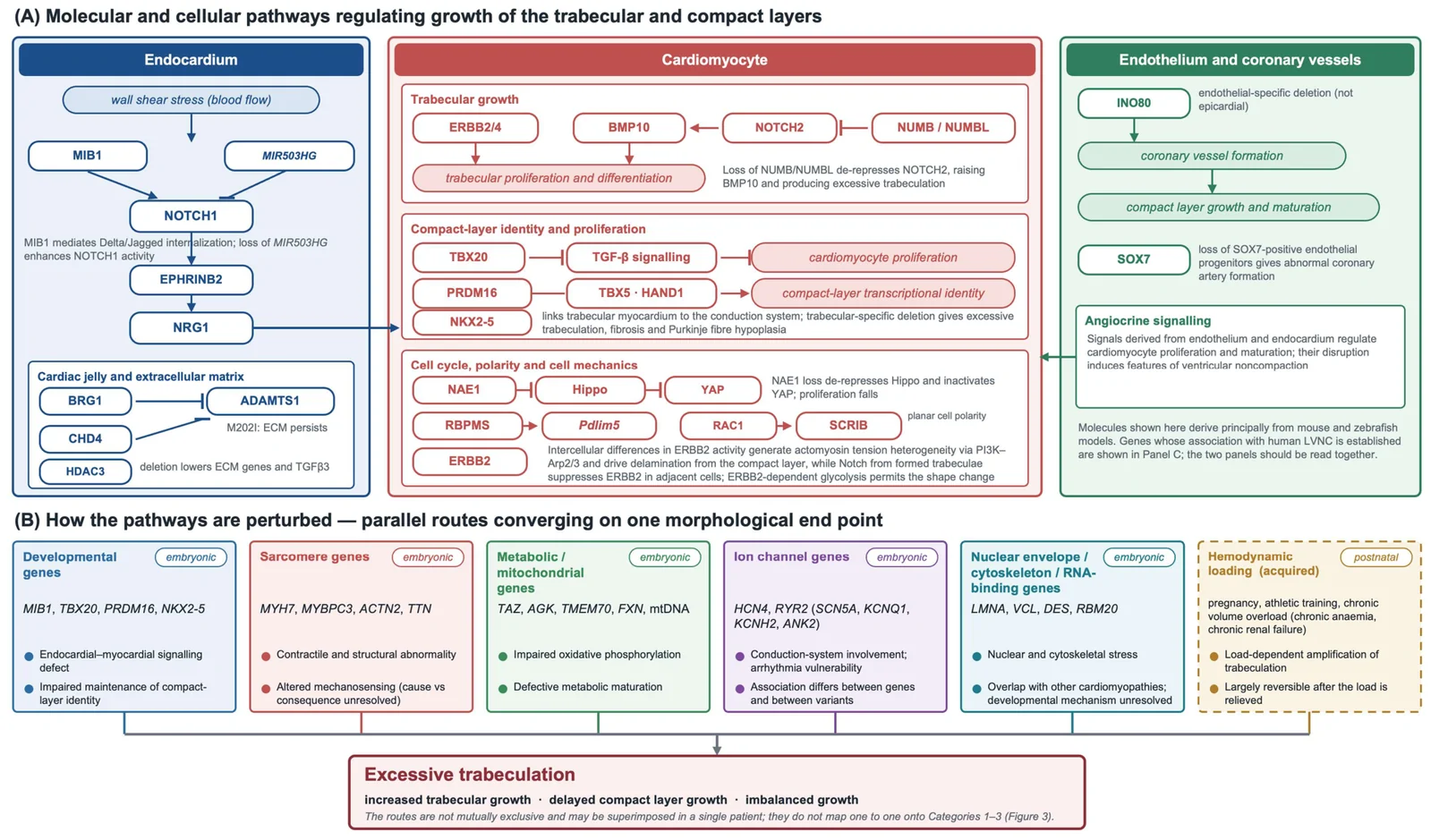
Molecular and cellular pathways of ventricular wall growth and the routes leading to excessive trabeculation. (**A**) The pathways that control trabeculation and growth of the compact layer: endocardial–myocardial signaling, transcriptional regulation, extracellular matrix and chromatin regulation, cell cycle, polarity and cell mechanics, and coordination with the coronary vasculature. (**B**) The six routes by which these pathways are perturbed—five genetic and one acquired, load-dependent—which act in parallel rather than in sequence and converge on the same morphology. The individual pathways, the genes assigned to each route and the supporting evidence are described in Section 3 and Section 4 and in Table 1 and are not repeated here. Unless otherwise indicated, the mechanisms shown derive from mouse, zebrafish or in vitro systems. ECM, extracellular matrix; LVNC, left ventricular noncompaction.

Rapid growth of the compact layer proceeds in coordination with formation of the coronary vasculature. In mice with endothelial-specific *Ino80* deletion, maturation of the ventricular wall and growth of the compact layer are impaired in association with defective coronary vascular formation [25], and single-cell analysis of the same model showed that angiocrine signals derived from the endothelium and endocardium regulate cardiomyocyte proliferation and maturation and that their disruption induces features of ventricular noncompaction [26]. Loss of *Sox7*-positive endothelial progenitor cells likewise produces abnormal coronary artery formation and impaired ventricular wall maturation [27]. The coronary vasculature therefore functions not merely as a route of oxygen supply but as a source of signals that drive compact layer formation (Figure 2A).

On the other hand, views differ regarding the fate of the intertrabecular recesses themselves. Lineage-tracing studies indicate that endocardium-derived cells can contribute to the coronary endothelium [18], whereas human morphometric studies do not support the compaction process itself [6,8], and the intertrabecular recesses persist in the adult heart as anatomical structures that communicate with the ventricular cavity but not with the coronary circulation [3,28]. The classical description that “intertrabecular spaces are remodeled into the microvasculature” therefore cannot be asserted at present, and this review confines itself to stating that “growth of the compact layer proceeds in coordination with intramyocardial ingrowth of coronary vessels and angiocrine signaling”.

The conclusion is most strongly supported in the human heart. In the mouse, a limited coalescence process cannot be entirely excluded, and in the chicken a marked increase in the compact layer with a reciprocal decrease in the trabecular layer is clearly observed, so oversimplification across species should be avoided [6]. Consistent with the differential growth model, the degree of trabeculation behaves in the general population as a continuous quantitative trait influenced by both common and rare variants rather than as a binary morphology [15], which is one basis for the use of the descriptive term “excessive trabeculation” in place of “noncompaction” [3].

## 3. Pathogenetic Mechanisms Leading to Excessive Trabeculation

The knowledge of normal ventricular wall formation makes it unlikely that the excessive trabeculation seen in LVNC arises from a single developmental defect. Endocardial–myocardial signaling, transcriptional regulation, the extracellular matrix, the cell cycle, polarity and mechanics, cardiomyocyte-intrinsic contractile and metabolic abnormalities, and postnatal hemodynamic loading may each be involved at different stages and converge on the same morphological end point. In the following, the hierarchy of evidence is distinguished with attention to whether a given finding derives from human genetic epidemiology, human tissue and imaging, mouse, zebrafish, or induced pluripotent stem cell-derived cardiomyocytes (iPSC-CMs). It should be noted that an established gene–disease association with LVNC is not synonymous with a verified mechanism by which abnormality of that gene product produces excessive trabeculation. This chapter addresses the latter, that is, the level at which mechanisms have been verified, whereas the evaluation of gene–disease validity itself is discussed in Section 4. Throughout this section, mechanisms demonstrated only in mouse, zebrafish or in vitro systems are identified as such; unless stated otherwise, the human relevance of these mechanisms has not been established.

### 3.1. Endocardial–Myocardial Signaling and Transcriptional Regulation

Trabeculation and maturation of the ventricular wall are controlled by bidirectional signaling between the endocardium and myocardium (Figure 2A; mainly demonstrated in mouse and zebrafish). Wall shear stress derived from embryonic blood flow acts as a required upstream input to this signaling [29]. The central pathway is Notch signaling. NOTCH1 activation in the endocardium induces *NRG1* expression via EphrinB2, and NRG1 activates myocardial ErbB2/ErbB4 to promote proliferation and differentiation of trabecular cardiomyocytes, while BMP10, which is enriched in the trabecular myocardium, maintains cardiomyocyte proliferation [16,30]. NOTCH1 and NRG1 respectively control degradation and synthesis of the cardiac jelly, and the balance between them defines the building plan for trabeculation [17].

Importantly, Notch signaling does not follow a simple relationship in which “reduction equals abnormality”. In humans, germline variants in *MIB1*, which encodes the E3 ubiquitin ligase responsible for internalization of the Notch ligands Delta/Jagged, are established as a cause of autosomal-dominant LVNC, and reduced NOTCH1 activity and target gene expression have been confirmed in affected individuals [31]. Conversely, in mice, loss of Numb/Numblike-mediated repression of Notch2 produces excessive Notch activity and elevated BMP10 and likewise causes excessive trabeculation [32]. In mice lacking the long non-coding RNA *MIR503HG*, enhanced Notch1 activity is likewise accompanied by thinning of the compact layer, excessive trabeculation, and diastolic dysfunction [33]. Thus, during development, the requirement is not the absolute level of Notch activity but maintenance of an appropriate range in terms of timing, cellular compartment, and intensity.

At the level of transcriptional regulation, PRDM16 is positioned as a factor that maintains the identity of compact-layer cardiomyocytes. In mice with cardiomyocyte-specific *Prdm16* deletion, left ventricular cardiomyocytes that should form the compact layer shift toward a trabecular-like transcriptional profile, and the left ventricle-specific program coordinated with TBX5 and HAND1 breaks down [24]. In patient-derived iPSC-CMs and knock-in mice carrying a *PRDM16* nonsense variant (Q187X), hypoplasia of the compact layer with reduced proliferation, increased apoptosis, and abnormal TGF-β signaling was reproduced [34]. *PRDM16* is also a candidate gene for the cardiomyopathy associated with 1p36 deletion syndrome, and variants are identified in non-syndromic LVNC and dilated cardiomyopathy as well [35]. *TBX20* controls the expression of regulators of TGF-β signaling, including *PRDM16*, and in patient-derived iPSC-CMs carrying *TBX20* variants, enhanced TGF-β signaling and reduced cardiomyocyte proliferation were observed, with improvement of the phenotype after TGF-β inhibition or genome-editing correction [36]. NKX2-5 controls the development of the trabecular myocardium and the conduction system, and its trabecular myocardium-specific deletion produces excessive trabeculation, fibrosis, and Purkinje fiber hypoplasia [37].

### 3.2. Extracellular Matrix, Chromatin, Cell Cycle, Polarity, and Cell Mechanics

It has further become clear that the extracellular matrix (ECM) and chromatin regulation are requirements for ventricular wall formation. In the mouse, the endocardial chromatin remodeling factor *Brg1* represses *Adamts1* to maintain the cardiac jelly and thereby permit trabeculation [38]. In mice engineered to model the human *CHD4* missense variant (M202I), enhanced CHD4–BRG1 interaction prevents appropriate *ADAMTS1* expression and produces biventricular excessive trabeculation through persistence of ECM and sustained cardiomyocyte proliferation; administration of ADAMTS1 corrected the phenotype [39]. Endocardial-specific *HDAC3* deletion in mice reduces ECM-related genes and *TGFβ3* and decreases trabeculation [40]. These findings indicate that the control of compact layer formation is not confined to primary abnormalities of transcription factors but constitutes a multilayered hierarchy that includes chromatin regulation and ECM dynamics.

The cell cycle, polarity, and cell mechanics are also determinants. Cardiomyocyte-specific deletion of the neddylation enzyme *NAE1* inactivates YAP through de-repression of the Hippo pathway, resulting in reduced cardiomyocyte proliferation and ventricular noncompaction [41]. In mice, deficiency of the RNA-binding protein RBPMS causes premature binucleation and cell cycle arrest in cardiomyocytes and produces a similar phenotype through aberrant splicing of *Pdlim5* [42]. In cardiomyocyte-specific *Rac1* deletion, cell shape and orientation are lost, and thinning of the compact layer and excessive trabeculation occur together with reduced levels of the planar cell polarity-related protein Scrib [43]. Recent live-imaging studies have proposed a model in which intercellular differences in ErbB2 activity generate heterogeneity of actomyosin tension via PI3K–Arp2/3 and induce delamination of cardiomyocytes from the compact layer toward the lumen, while Notch signaling from the formed trabeculae suppresses ErbB2 activity in adjacent cells and maintains their compact-layer fate [44]. In addition, wall shear stress derived from embryonic blood flow acts as a signal required for trabeculation via endocardial Notch/NRG1 signaling [29], and ErbB2-dependent enhancement of glycolysis enables the morphological changes of cardiomyocytes [45]. Trabeculation is therefore determined not by the amount of proliferation but by the spatiotemporal coordination of proliferation, differentiation, ECM metabolism, cell mechanics, and metabolic maturation.

### 3.3. Pathways by Which Cardiomyocyte-Intrinsic Abnormalities Interfere with Developmental and Maturation Programs

Sarcomere gene variants are frequently identified in LVNC, but the underlying mechanisms are not as straightforward as those of developmental genes (Figure 2B). Large-scale rare variant analyses have shown that protein-truncating variants in *MYH7*, *ACTN2*, and *PRDM16* are relatively specifically enriched in LVNC [13], and in a Japanese fetal-onset cohort up to 75% of the pathogenic variants identified were sarcomere-related [21]. In patient-derived iPSC-CMs, altered expression of cardiac developmental genes has been reported in addition to abnormalities in sarcomere structure and Ca2+ handling [46]; this is an in vitro observation in a patient-derived model, and it suggests that the sarcomere may function not only as the postnatal contractile apparatus but also, during embryonic life, as a device that senses mechanical input and promotes maturation. In *MYH7* Q315R knock-in mice, an animal model, extensive metabolic remodeling of glycolysis, fatty acid oxidation, and the TCA cycle was observed in addition to excessive trabeculation and cardiac dysfunction [47]. However, whether these changes are the cause of the morphogenetic abnormality or the consequence of contractile dysfunction remains unresolved, and direct verification of how mechanical mechanisms mediated by reduced contractile force or altered wall stress modify embryonic trabecular growth is still at the level of hypothesis.

Genes encoding nuclear envelope, cytoskeletal, and RNA-binding proteins (such as *LMNA*, *VCL*, *DES*, and *RBM20*) have been reported in cases presenting with LVNC morphology and show extensive genetic overlap with dilated cardiomyopathy and arrhythmogenic cardiomyopathy [13,48,49]. However, experimental evidence directly supporting a mechanism by which abnormalities of these gene products interfere with embryonic ventricular wall formation is currently scarce. This review therefore limits itself to describing them as a genetic background shared with other cardiomyopathies that may be associated with LVNC morphology and positions their involvement in developmental mechanisms as unresolved.

A similar reservation is required for ion channel-related genes, and the strength of the association differs between genes and, within the same gene, between variants. For *HCN4*, variants in the pore or transmembrane region co-segregate with a combined phenotype of bradycardia and LVNC in multiple independent families, and functional analysis has demonstrated a dominant negative effect [50,51]. For *RYR2*, most carriers of the exon 3 deletion show LVNC morphology [52]. Enrichment of these variants has also been reported in LVNC cohorts [13], and in a Japanese multicenter cohort, patients positive for ion channel-related gene variants were reported to show biventricular excessive trabeculation together with conduction abnormalities and arrhythmia burden despite preserved left ventricular systolic function [53]. By contrast, *SCN5A*, *KCNQ1*, *KCNH2* and *ANK2* have established associations with primary arrhythmia syndromes, whereas their association with LVNC morphology is limited at the gene level. Even for the variants for which the association is comparatively supported, experimental evidence demonstrating a mechanism by which channel dysfunction itself generates excessive trabeculation is scarce. Neither the possibility that these findings reflect the close developmental relationship between the trabecular myocardium and the conduction system [37] nor the possibility that a primary channelopathy and left ventricular hypertrabeculation coexist incidentally can be excluded, and at present the mechanism should be positioned as hypothetical.

Metabolic and mitochondrial abnormalities likewise require a distinction between primary etiology and secondary change. TAZ mediates cardiolipin remodeling, and its deficiency impairs mitochondrial inner membrane structure and oxidative phosphorylation, causing Barth syndrome, which may present with an LVNC or DCM-like phenotype [54,55]. Impaired oxidative phosphorylation due to abnormalities of nuclear DNA or mtDNA has also been reported in association with HCM, DCM, RCM, and LVNC [56,57]. Because the embryonic compact layer has high proliferative activity and energy demand, a link is postulated between the metabolic maturation of cardiomyocytes (the transition from glycolysis-dominant metabolism to fatty acid oxidation) and growth of the compact layer. Human evidence for this causal relationship is, however, limited.

### 3.4. Load-Dependent and Reversible Excessive Trabeculation

There is, in addition, a pathway by which postnatal hemodynamic loading and myocardial remodeling amplify trabeculation in an acquired manner (Figure 2B). In longitudinal evaluation of primiparous women who were morphologically normal at baseline, an increase in trabeculation was observed during pregnancy and some women met the imaging diagnostic criteria for LVNC, but at postpartum follow-up, most of these changes had disappeared or markedly regressed [9]. Similar increases in trabeculation have been reported in athletes undergoing high-intensity training [10]. Increased trabeculation has also been reported in conditions with chronic volume loading such as chronic anemia and chronic renal failure [28]. These human observational studies indicate that at least a portion of excessive trabeculation can appear in an acquired and reversible manner.

This phenomenon can be understood as an adaptive response of the ventricular wall to increased preload and cardiac output. Possible molecular mechanisms include the myocardial stretch response and partial reactivation of the fetal gene program, but molecular verification in humans is insufficient, and these remain hypothetical. It is not appropriate to describe such load-dependent changes as “the onset of acquired LVNC”; they should be described as load-dependent hypertrabeculation, a phenomenon in which trabeculation is augmented in response to loading, and distinguished from inherited and developmental cardiomyopathy [3]. Furthermore, no prospective data exist on whether mechanical loading increases penetrance in carriers of pathogenic variants.

### 3.5. The Myocardial Tissue Substrate Demonstrated by Pathology and CMR

In autopsy specimens and hearts explanted at transplantation, LVNC is observed as a two-layered structure with a thin compact layer and deep intertrabecular recesses. Histologically, endocardial fibrosis or findings resembling endocardial fibroelastosis are most frequently reported, and the myocardium shows predominantly subendocardial degeneration and up to moderate fibrosis, whereas the extensive fibrotic foci typically seen in dilated cardiomyopathy are sparse [28]. Whether endocardial fibrosis is due to immunological mechanisms, to local flow abnormalities, or to hemodynamic and mechanical factors around the trabeculae is unclear [28]. Subendocardial hypoperfusion and reduced coronary flow reserve in patients without coronary artery stenosis have been hypothesized to contribute to systolic dysfunction [28].

Tissue characterization by CMR complements this pathological picture non-invasively. Late gadolinium enhancement (LGE) reflects focal fibrosis, whereas native T1 mapping and the extracellular volume fraction (ECV) reflect diffuse changes in tissue characteristics. In patients with LVNC, native T1 and ECV were elevated relative to controls even in LGE-negative cases (native T1 1024 ± 43 vs. 995 ± 22 ms at [field strength] using a [sequence] sequence; ECV 28.0 ± 4.5% vs. 23.5 ± 2.2%), which may reflect diffuse myocardial tissue changes not captured by LGE [58]. Native T1 values are not comparable across field strengths, sequences or vendors and should be interpreted against site-specific reference ranges.

With regard to prognosis, LGE and global systolic impairment are important risk indicators [59], and in a multicenter study of 585 patients, the left ventricular ejection fraction was a strong predictor of major adverse cardiac events [60]. In a multicenter CMR study of 214 adults, major adverse cardiac events were more frequent in patients with compact myocardial thinning of 50% or more relative to adjacent segments (hazard ratio 3.05, 95% confidence interval 1.57–5.94), and the association persisted in multivariable analysis [61]. Another adult CMR study likewise found that thinning of the compact layer was associated with lower systolic function [62]. These findings suggest that clinical functional impairment may be determined not by the amount of trabeculation itself but by quantitative and qualitative abnormalities of the compact layer and by myocardial tissue characteristics, including fibrosis. All of these studies, however, demonstrate association rather than causation, and the evidence is insufficient to regard compact layer thinning as an LVNC-specific etiological marker or as an obligatory diagnostic criterion.

Furthermore, in *MIB1*-deficient LVNC mice, connexin43 is redistributed from the intercalated discs to the lateral membrane, with abnormalities of action potential duration and repolarization [63]. This is an experimental finding that links developmental and morphological abnormality with electrical remodeling, but caution is required in extrapolating it directly to arrhythmic risk in humans.

## 4. Genetic Heterogeneity

### 4.1. The Hierarchy of Evidence for Gene–Disease Validity

Many genes have been reported in association with LVNC, but having been reported is not synonymous with having an established causal relationship. In a systematic analysis of 189 genes using the ClinGen evaluation framework, only 11 genes (6%) were rated as having “definitive” disease association with LVNC and 21 (11%) as “moderate”, whereas 140 (74%) were rated “limited” and 17 (9%) as having “no evidence” [14]. In other words, the great majority of reported genes have not reached a level of gene–disease validity at which their molecular mechanisms can be meaningfully discussed (Table 1). Genetic interpretation therefore requires separate evaluation of gene–disease validity, the pathogenicity of the individual variant, and consistency with the patient’s phenotype. Evidence is moreover not uniform at the level of the gene: within the same gene, validity may differ between variants (see Section 4.3 and Section 4.4). In the following, genes are organized for convenience—on the basis of pathogenetic mechanism and clinical features—into sarcomeric, developmental, ion channel-related, and metabolic and mitochondrial groups, together with genes spanning multiple functional categories.

### 4.2. Sarcomere Genes

Sarcomere gene variants are the most frequent genetic cause of LVNC, accounting for 25–30% of the P/LP variants identified in international cohorts—a proportion of the variants identified, not of all patients [11,12,13,14]. In an analysis comparing 840 patients with LVNC and 125,748 gnomAD controls, truncating variants in *MYH7* were enriched approximately 20-fold in LVNC relative to controls, and this enrichment was not observed in hypertrophic or dilated cardiomyopathy cohorts [13]. *MYH7* is the most frequent gene, followed by *MYBPC3*, *ACTC1*, *TPM1*, and *TNNT2*. In a Japanese multicenter cohort study, patients positive for P/LP variants had an earlier age at onset, a lower left ventricular ejection fraction at diagnosis, and a higher frequency of the dilated cardiomyopathy phenotype and of death or cardiac transplantation [12]. In the long-term cohort study of van Waning et al., by contrast, patients carrying multiple P/LP variants and those carrying *TTN* variants had a higher risk of left ventricular systolic dysfunction and of adverse cardiac events, whereas patients positive for *MYH7* variants experienced fewer such events [64]. Patients with sarcomere variants therefore cannot be treated as a uniform group with respect to outcome, and evaluation must be performed gene by gene and according to variant burden. In fetal-onset LVNC, sarcomere gene variants have been reported to account for up to 75% of the P/LP variants identified [21], suggesting the existence of mechanisms by which the morphological phenotype is established from embryonic life.

### 4.3. Developmental Genes

Developmental genes are linked to molecular pathways that directly control ventricular wall patterning and trabeculation. Loss-of-function variants in *MIB1*, a regulator of the Notch pathway, are strongly supported as causal for LVNC, with both co-segregation in human families and reproduction of the phenotype in mouse models [31]. For *TBX20* as well, a large-scale analysis in 2024 identified protein-truncating variants in 24 (0.32%) of 7463 DCM/LVNC probands, representing marked enrichment relative to internal controls (0.004%) and gnomAD (0.003%), with co-segregation demonstrated in 21 families, substantially strengthening the gene–disease association [65]. *PRDM16* is important in that human genetic enrichment [13], 1p36 deletion syndrome [35], patient-derived iPSC-CMs and knock-in mice [34], and the molecular mechanism of maintaining compact-layer cardiomyocyte identity [24] all converge on this gene.

*BMP10* requires a distinction between two kinds of evidence. It is a central developmental signaling molecule, specifically expressed in the trabecular myocardium of the embryonic heart and indispensable in model animals for maintaining cardiomyocyte proliferation [30]. In humans, screening of familial LVNC identified the rare variant p.V407I, which was also present in affected family members; the mutant protein showed reduced binding to the BMP receptors BMPR1a and BMPR2 and, in cardiomyoblasts, reduced proliferation and reduced tolerance to stretch stimulation [66]. The number of reported families is nevertheless small, and independent replication is scarce; thus, gene–disease validity remains limited relative to that of *MIB1*, *TBX20*, or *PRDM16* [14], and *BMP10* is best interpreted as a candidate gene. Being a central molecule of a developmental pathway and being an established causal gene for human LVNC should therefore be described separately.

### 4.4. Ion Channel-Related Genes

Ion channel-related genes cannot be treated as a single group with respect to LVNC. Variants in the pore or transmembrane region of *HCN4* co-segregate with bradycardia and LVNC in multiple independent families with supporting functional data [50,51], and most carriers of the *RYR2* exon 3 deletion show LVNC morphology [52]; the enrichment of both has also been reported in LVNC cohorts [13]. For *SCN5A*, *KCNQ1*, *KCNH2* and *ANK2*, by contrast, the association with primary arrhythmia syndromes is established, whereas the association with LVNC morphology is limited at the gene level. In a Japanese multicenter cohort, patients positive for ion channel-related gene variants were reported to show biventricular excessive trabeculation, conduction abnormalities, and arrhythmia burden despite relatively preserved left ventricular systolic function [53]. A causal mechanism by which channel gene abnormalities themselves generate excessive trabeculation has nevertheless not been established, and interpretation must proceed gene by gene and variant by variant, distinguishing an electromechanical overlap phenotype, in which a single genetic defect produces both the arrhythmic and the structural phenotype, from incidental coexistence of a primary arrhythmia syndrome with left ventricular hypertrabeculation (Section 7.4).

### 4.5. Metabolic and Mitochondrial Genes

In mitochondrial disease, oxidative phosphorylation is impaired by abnormalities of nuclear DNA or mtDNA, and hypertrophic, dilated, and restrictive cardiomyopathy as well as LVNC may result [56,57]. *TAZ* variants are the cause of Barth syndrome and are representative of the metabolic and mitochondrial group in LVNC. In reports from Japan, cases with *TAZ* variants were characterized by a dilated cardiomyopathy phenotype in early infancy and rapid clinical deterioration, and the clinical phenotypes of variants within a family ranged remarkably widely, from severe infantile dilated cardiomyopathy to mild adult LVNC [54,55]. When a *TAZ* variant is identified, multidisciplinary evaluation including hematological assessment for neutropenia and metabolic screening for 3-methylglutaconic aciduria is essential. In addition to *TAZ*, *AGK*, *TMEM70*, *FXN*, and mtDNA variants have been reported as etiologies associated with LVNC [56,57]. When a metabolic or mitochondrial etiology is suspected, measurement of blood lactate and urinary organic acids and multiorgan evaluation including the nervous system, skeletal muscle, liver, kidney, and endocrine system should be performed, and genetic counseling taking maternal or X-linked inheritance into account should be provided [67].

### 4.6. Genetic Overlap with Other Cardiomyopathies and Multiple Variants

The four groups above constitute a convenient organization based on pathogenetic mechanism and are not a mutually exclusive classification. Some genes span multiple functional categories; for example, *TTN* and *ACTN2* are involved in sarcomere structure and at the same time show extensive genetic overlap with other cardiomyopathies, including dilated cardiomyopathy (DCM). Furthermore, nuclear envelope proteins (*LMNA*), cytoskeletal proteins (*VCL*, *DES*), and RNA-binding proteins (*RBM20*) have also been reported in cases presenting with LVNC morphology [13,48]. The mechanisms by which these produce excessive trabeculation are, however, unresolved (see Section 3.3). These genes are shared with dilated and arrhythmogenic cardiomyopathy, suggesting that LVNC morphology is less a distinct single disease than a morphological finding that may appear in common across multiple cardiomyopathies [49].

In patients carrying multiple P/LP variants, associations with severe phenotypes and with death or cardiac transplantation have been reported in selected cohorts [12,53,64]. Their independent predictive value has not been established, however, and the level of risk cannot be judged from variant number alone. Genotype should be interpreted in integration with age at onset, ventricular function, arrhythmia, family history, and clinical phenotype.

**Table 1:** Gene groups associated with LVNC: gene–disease validity, postulated mechanism, level of mechanistic verification, clinical utility, and relationship to the framework.

Functional Group	Representative Genes	Gene–Disease Validity for LVNC	Postulated Mechanism of Excessive Trabeculation	Level at Which the Mechanism Has Been Verified	Clinical Utility for Outcome Assessment	Relationship to the Framework (Category 1 or Risk Modifier Within Category 3)
Developmental	*MIB1*, *TBX20*, *PRDM16*, *NKX2-5*; (*BMP10*, *NOTCH1*)	Highest among the groups, but not uniform. *MIB1*: moderate—co-segregation in human families with reproduction of the phenotype in mice [31]. *TBX20*: truncating variants in 24 of 7463 DCM/LVNC probands (0.32%) vs. 0.004% internal controls and 0.003% gnomAD, with cosegregation in 21 families [65]. *PRDM16*: human genetic enrichment [13] and 1p36 deletion syndrome [35]. *BMP10* and *NOTCH1*: candidate—experimental and functional support exists, but gene–disease validity remains limited [14,66].	Primary disturbance of endocardial–myocardial (NOTCH1–NRG1–ErbB) signaling, of maintenance of compact-layer cardiomyocyte identity, and of TGF-β signaling [17,24,31,36].	Highest. Human genetics, mouse models, and patient-derived iPSC-CMs converge on the same mechanism [24,31,34,36].	Investigational. Reported case numbers are small, and no association with a defined outcome has been established.	Category 1 when an overt functional abnormality is present. When an overt cardiomyopathy-level functional abnormality is absent, the same genetic finding does not indicate Category 1 but constitutes a risk modifier within Category 3.
Sarcomere	*MYH7*, *MYBPC3*, *ACTC1*, *TPM1*, *TNNT2*, *ACTN2*, *TTN*	*MYH7*: definitive. *MYBPC3*, *ACTC1*, *TPM1*, *TNNT2*: moderate to limited. Most frequent genetic cause, accounting for 25–30% of the P/LP variants identified in international cohorts—a proportion of variants, not of patients [11,12,13,14]. Truncating *MYH7* variants are enriched approximately 20-fold over gnomAD and are not enriched in HCM or DCM cohorts [13]. Up to 75% of P/LP variants in fetal-onset disease [21].	Unresolved whether the structural and contractile abnormality is the cause of the morphogenetic defect or its consequence. A role for the sarcomere as an embryonic mechanosensing and maturation device is hypothesized [46].	Human genetic epidemiology strong; mechanism at the level of hypothesis, based on iPSC-CMs [46] and a single knock-in mouse line [47].	Suggestive. The direction differs by gene: *MYH7*-positive patients do not necessarily experience more adverse cardiac events [64].	Category 1 when an overt functional abnormality is present. When an overt cardiomyopathy-level functional abnormality is absent, the same genetic finding does not indicate Category 1 but constitutes a risk modifier within Category 3.
Ion channel	*HCN4*, *RYR2*, *SCN5A*, *KCNQ1*, *KCNH2*, *ANK2*	Variable, and differing at the variant level. Comparatively supported: pore and transmembrane variants of *HCN4*, which co-segregate with bradycardia and LVNC in multiple independent families with functional evidence of a dominant-negative effect [50,51], and the *RYR2* exon 3 deletion, most carriers of which show LVNC morphology [52]; enrichment of both also reported [13]. Limited at gene level: *SCN5A*, *KCNQ1*, *KCNH2*, *ANK2*, whose established associations are with primary arrhythmia syndromes rather than with LVNC.	No experimental evidence that channel dysfunction itself generates excessive trabeculation. May reflect the close developmental relationship between trabecular myocardium and the conduction system [37], or incidental coexistence of a primary channelopathy with hypertrabeculation.	Human cohort observation only; the mechanism remains hypothetical.	Suggestive. Useful for arrhythmia evaluation; not established as a predictor of a defined outcome.	Category 1 (arrhythmia-predominant phenotype) where the arrhythmic and structural phenotypes are pathophysiologically related—an electromechanical overlap phenotype. Where they are explained by an independent primary arrhythmia syndrome, Category 3 with the arrhythmia syndrome managed as a comorbid disease.
Metabolic and mitochondrial	*TAZ*, *AGK*, *TMEM70*, *FXN*, mtDNA	*TAZ*: definitive as the cause of Barth syndrome [54,55]. Others: limited; reported as LVNC-associated etiologies [56,57].	Impaired cardiolipin remodeling compromises mitochondrial inner membrane structure and oxidative phosphorylation [54,55]; impaired oxidative phosphorylation also arises from nuclear DNA or mtDNA abnormalities [56,57]. A link between cardiomyocyte metabolic maturation and compact layer growth is postulated.	Biochemical mechanism established; the developmental mechanism linking metabolic maturation to compact layer growth is not verified in humans.	Suggestive. Associated with early-onset severe presentation and with death or cardiac transplantation.	Category 1 when an overt functional abnormality is present. When an overt cardiomyopathy-level functional abnormality is absent, the same genetic finding does not indicate Category 1 but constitutes a risk modifier within Category 3.
Nuclear envelope, cytoskeleton, RNA-binding	*LMNA*, *VCL*, *DES*, *RBM20*	Limited. Reported in cases presenting with LVNC morphology [13,48], with extensive overlap with dilated and arrhythmogenic cardiomyopathy [49].	Unresolved. No direct experimental evidence supports interference with embryonic ventricular wall formation (Section 3.3).	Human case reports and cohorts only; no developmental mechanistic data.	Investigational for LVNC specifically. Best regarded as a genetic background shared across cardiomyopathies [49].	Category 1 when an overt functional abnormality is present. When an overt cardiomyopathy-level functional abnormality is absent, the same genetic finding does not indicate Category 1 but constitutes a risk modifier within Category 3.
Multiple P/LP variants	(not gene-specific)	Not applicable—a risk modifier rather than a gene–disease relationship.	Not applicable.	Not applicable.	Not established. Associations with severe phenotypes and with death or cardiac transplantation are reported in selected cohorts [12,53,64], but independent predictive value has not been shown.	Does not by itself determine either the category or the level of risk.

DCM, dilated cardiomyopathy; HCM, hypertrophic cardiomyopathy; iPSC-CM, induced pluripotent stem cell-derived cardiomyocyte; LVEF, left ventricular ejection fraction; LVNC, left ventricular noncompaction; P/LP, pathogenic or likely pathogenic. Gene–disease validity refers to the association with LVNC specifically. Several genes listed have established associations with arrhythmia syndromes or with other cardiomyopathies rather than with LVNC. Evidence may differ at the variant level as well as at the gene level. In a systematic ClinGen-based assessment of 189 genes, only 11 (6%) were rated definitive and 21 (11%) moderate for disease association with LVNC, whereas 140 (74%) were limited and 17 (9%) had no evidence [14]. The functional grouping is a convenience based on pathogenetic mechanism and is not mutually exclusive; *TTN* and *ACTN2*, for example, span more than one category. Gene–disease validity and the level at which a mechanism has been verified are separate axes and are reported in separate columns. Validity assignments should be reconfirmed against the current ClinGen curation before submission.

## 5. Diversity of Clinical Phenotypes

Genetic heterogeneity is observed clinically not as a simple one-to-one correspondence but as diverse phenotypes. Synthesizing findings from pediatric LVNC cohorts, representative phenotypic patterns can be described, for clinical convenience, as the following seven groups (Table 2) [12,53,68,69,70]. These are descriptions based on different axes—systolic and diastolic function, wall thickness, arrhythmia, anatomical distribution, and comorbidity—and are not a mutually exclusive disease classification; they are descriptive designations and do not correspond one to one with Categories 1–3 (Section 7.4). The designations used below denote states in which another cardiomyopathy phenotype coexists with excessive trabeculation; they do not imply a subclassification with LVNC as the parent category. These groups derive from several pediatric cohort reports whose study populations, diagnostic criteria, and registry formats differ, so it is not appropriate to combine and present a frequency for each group; the groups may also overlap with one another. Where relative frequency is relevant, it is stated qualitatively.

Outcomes are reported in this review in four categories: (1) death or cardiac transplantation, (2) worsening heart failure, (3) ventricular arrhythmia and sudden-death-related events, and (4) thromboembolism. Composite expressions such as “poor prognosis” or “high risk” are avoided, and the relevant outcome is specified instead.

### 5.1. Representative Clinical Phenotypic Patterns

1. Hypertrabeculation with preserved cardiac function: systolic function is preserved, patients are asymptomatic without clinically significant arrhythmia, and the condition is detected through family screening or incidental testing. The detection rate of P/LP variants is lower than in the other groups [12,68].
2. DCM phenotype with hypertrabeculation: the most frequent phenotypic group, presenting with left ventricular dilatation and reduced systolic function. The risk of death or cardiac transplantation is higher than in the other phenotypic groups; sarcomere gene variants including *MYH7* are enriched in some cohorts, with earlier onset and more severe reduction in LVEF [12,53,68].
3. Hypertrophic cardiomyopathy (HCM) phenotype with hypertrabeculation: an overlap phenotype in which left ventricular wall thickening and excessive trabeculation coexist, with reported associations with *MYBPC3* or *MYH7* variants [12,68]. The clinical course is largely determined by the HCM phenotype dominated by wall thickening. This is the situation in which the attribution judgement is unavoidable: in two asymptomatic adolescents who satisfied both the hypertrophic cardiomyopathy criteria and the Petersen criterion simultaneously, imaging alone could not establish whether the trabeculation exceeded what the hypertrophy would explain [71]. Where the temporal sequence is also unavailable, such cases are recorded as not yet assigned between Categories 1 and 2 (Section 7.2).
4. Restrictive cardiomyopathy (RCM) phenotype with hypertrabeculation: among the rarest phenotypic groups, in which a restrictive filling pattern and excessive trabeculation coexist, and which may follow a severe course. In patients with infantile onset and rapidly progressive diastolic dysfunction, early evaluation for cardiac transplantation should be considered [68].
5. Arrhythmia-predominant phenotype with hypertrabeculation: clinically important arrhythmias occur despite preserved or only mildly reduced systolic function. Variants for which the association with LVNC morphology is comparatively supported, such as those in the pore or transmembrane region of *HCN4* and the *RYR2* exon 3 deletion, have been reported [13,50,51,52]; conversely, this group may also include cases in which excessive trabeculation coexists incidentally with a primary arrhythmia syndrome, so the pathophysiologic relatedness of the arrhythmic and the myocardial phenotype must be assessed individually [53].
6. Hypertrabeculation involving both ventricles (reported as biventricular noncompaction, BiVNC): characterized by excessive trabeculation of both ventricles, frequently diagnosed in the perinatal period and associated with a higher risk of death or cardiac transplantation. In a Japanese cohort, sarcomere genes were most frequent, whereas the proportion of mitochondrial and developmental gene variants was higher than in other phenotypic groups [70].
7. Hypertrabeculation associated with congenital heart disease (CHD): coexisting structural cardiac malformation independently increases the risk of worsening heart failure. Variants in genes involved in developmental pathways (*BMP10*, *NKX2-5*) have been reported in some cases, but gene–disease validity must be evaluated individually [69].

### 5.2. Integrated Interpretation of Genetic Information, Age at Onset, and Outcome

Taken together, the published data indicate that both phenotypic group and genotype are associated with outcome. By phenotype, the DCM phenotype and hypertrabeculation involving both ventricles carry a higher risk of death or cardiac transplantation [68,70]. Age at onset is an important determinant of these phenotypic groups: fetal and infantile onset are associated with a higher frequency of severe phenotypic groups and a higher proportion of patients positive for P/LP variants [21,22,72]. By genotype, death or cardiac transplantation and worsening heart failure have been reported more frequently in the metabolic and mitochondrial group and in patients carrying multiple P/LP variants [12,54,64], whereas in the group with a single sarcomere variant the association differs by gene, and patients positive for *MYH7* variants do not necessarily experience more of these events [64]. Moreover, patients negative for P/LP variants do not necessarily follow an uneventful course, since the current genetic diagnostic yield is limited, and a negative finding does not mean the absence of genetic predisposition [11,12,14]. Genetic information thus adds to outcome assessment but cannot by itself determine the course of an individual patient.

Importantly, these seven phenotypic patterns do not belong to the same conceptual level as the three clinical pathophysiologic contexts described below. The phenotypic groups are a descriptive account based on morphological and functional findings (Axis 2), whereas the three contexts are a judgement about standing as a disease entity (Axis 1). Because the same patient may satisfy more than one phenotypic group, a category cannot be determined unequivocally from the phenotypic group; even within the same DCM phenotype, HCM phenotype, biventricular involvement, or arrhythmia-predominant phenotype, the pathophysiologic position may differ according to underlying disease, pathological hemodynamic loading, genetic background, timing of onset, and change over time. Phenotypic pattern and pathophysiologic context must therefore be considered separately.

**Table 2:** Clinical phenotypic groups of excessive trabeculation: principal postulated mechanism, corresponding pathophysiologic context, and recommended evaluation.

Clinical Phenotypic Group (Axis 2)	Principal Postulated Mechanism	Category Mainly Corresponding (Axis 1)	Recommended Evaluation
(1) Hypertrabeculation with preserved cardiac function	Physiological variation, or the preclinical phase of a genetic predisposition. Detection rate of P/LP variants is lower than in the other groups [12,68].	Category 3, stratified by the presence or absence of risk modifiers.	Three-generation family history and screening of first-degree relatives by echocardiography and electrocardiography. Subsequent follow-up is conditional (Section 7.2): where an evident physiological trigger is present, no modifier is identified and the findings regress, cardiomyopathy-level follow-up is not required; where neither trigger nor modifier is evident, morphology, function and the electrocardiogram are re-evaluated at intervals; where risk modifiers are present, serial echocardiography and electrocardiography are performed at individualized and shorter intervals. A negative genetic test does not exclude genetic predisposition. These follow-up recommendations are expert proposals.
(2) DCM phenotype with hypertrabeculation	Interference with ventricular wall growth by sarcomeric or cytoskeletal abnormality. Sarcomere variants including *MYH7* are enriched in some cohorts, with earlier onset and lower LVEF [12,53,68].	Category 1 where the trabeculation precedes or accompanies the functional abnormality; Category 2 where an established DCM phenotype was documented before it appeared.	Assessment of cardiac function, genetic testing, heart failure management, and assessment of outcomes (1) and (2). The most frequent group, with a higher risk of death or cardiac transplantation [12,53,68].
(3) HCM phenotype with hypertrabeculation	Coexistence of excessive trabeculation with an underlying cardiomyopathy (HCM). Associations with *MYBPC3* or *MYH7* variants reported [12,68,71].	Category 1 where the trabeculation precedes or accompanies the functional abnormality; Category 2 where an established HCM phenotype was documented before it appeared; not yet assigned where the temporal sequence cannot be established (Section 7.2).	Evaluation and management according to HCM.
(4) RCM phenotype with hypertrabeculation	Myocardial abnormality dominated by diastolic impairment. Among the rarest groups; gene–disease relationships not established.	Category 1 where the trabeculation precedes or accompanies the functional abnormality; Category 2 where an established RCM phenotype was documented before it appeared; not yet assigned where the temporal sequence cannot be established (Section 7.2).	Assessment of diastolic function; early evaluation for cardiac transplantation where onset is infantile and diastolic dysfunction is rapidly progressive [68].
(5) Arrhythmia-predominant phenotype with hypertrabeculation	Arrhythmic substrate in trabecular myocardium, or an independent primary arrhythmia syndrome. Comparatively supported variants include pore and transmembrane variants of *HCN4* and the *RYR2* exon 3 deletion [13,50,51,52,53].	Category 1 where the arrhythmic and structural phenotypes are pathophysiologically related; Category 3 with a comorbid disease where coexistence is incidental (Section 7.4).	Electrocardiography, Holter monitoring, exercise testing, genetic testing, assessment of co-segregation of both phenotypes within the family, differentiation from a primary arrhythmia syndrome, and assessment of outcome (3).
(6) Hypertrabeculation involving both ventricles (reported as BiVNC)	Impaired wall formation of both ventricles through developmental signaling or metabolic abnormality. Sarcomere genes most frequent, with a higher proportion of mitochondrial and developmental variants than in other groups [70].	Category 1	Assessment of cardiac function from the perinatal period; metabolic and mitochondrial evaluation. Frequently diagnosed perinatally, with a higher risk of death or cardiac transplantation [70].
(7) Hypertrabeculation associated with congenital heart disease	Amplification of trabeculation by volume or pressure loading, with or without a coexisting developmental abnormality. Developmental pathway variants (*BMP10*, *NKX2-5*) reported in some cases [69].	Category 2 where the loading explains the trabeculation; reassessment after repair.	Management according to the underlying congenital heart disease; reassessment of the category after the loading is relieved. Coexisting structural malformation independently increases the risk of worsening heart failure [69].

Abbreviations as in Table 1; BiVNC, biventricular noncompaction; CHD, congenital heart disease; RCM, restrictive cardiomyopathy. These groups derive from several pediatric cohort reports whose study populations, diagnostic criteria and registry formats differ, so a frequency is not given for each group; the groups may also overlap with one another. The phenotypic group (Axis 2) is a descriptive account, whereas the category (Axis 1) is a judgement about standing as a disease entity; the two do not correspond one to one, and the category is determined individually from the presence of an overt functional abnormality and the availability of an alternative explanation (Section 7.4). Risk within a category is stratified separately by risk modifiers (Axis 3). Outcomes are numbered as defined in Chapter 5: (1) death or cardiac transplantation, (2) worsening heart failure, (3) ventricular arrhythmia and sudden-death-related events, (4) thromboembolism.

## 6. Why a Conventional Single-Disease Concept or Single Axis Cannot Organize This Entity

Integrating normal development, pathogenetic mechanisms, genetics, and clinical phenotypes as reviewed above reveals structural limitations in defining LVNC by a single morphological criterion, a single genetic background, or a single clinical phenotype. The problem is that the common morphological end point of excessive trabeculation encompasses developmental cardiomyopathy, changes accompanying other cardiac diseases, load-dependent adaptation, and latent preclinical states.

### 6.1. Limitations of Morphology and of the Term “Noncompaction”

The 2023 JACC Cardiovascular Imaging expert panel paper proposed use of the descriptive term “excessive trabeculation” rather than “noncompaction”, on the grounds that the developmental premise that trabeculae coalesce into the compact layer in humans is not supported by sufficient morphometric evidence [3]. The criteria at issue are those of Jenni et al., an end-systolic noncompacted-to-compacted (N/C) ratio greater than two on echocardiography [20]; Petersen et al., an end-diastolic N/C ratio greater than 2.3 on cardiac magnetic resonance [73]; and Jacquier et al., a trabeculated left ventricular mass exceeding 20% of global left ventricular mass on cardiac magnetic resonance [74]. In this review, an N/C ratio greater than two refers throughout to the echocardiographic end-systolic criterion, and an N/C ratio greater than 2.3 to the cardiac magnetic resonance end-diastolic criterion. These criteria differ in modality, cardiac phase and denominator and are not interchangeable; all were derived in adults, and none has been validated against outcomes in children. Prominent normal trabeculation, false tendons, apical thrombus and load-related trabeculation should be distinguished from the finding under discussion before any criterion is applied. The 2023 ESC Guidelines on cardiomyopathies likewise do not regard left ventricular hypertrabeculation as a distinct cardiomyopathy in the general sense, treating it instead as a morphological trait that may occur in isolation or together with other developmental abnormalities, wall thickening, chamber dilatation, or systolic dysfunction [4]. This position is consistent with human developmental biology supporting differential growth [6,7,8], with genetic and imaging studies showing that trabeculation is a continuous quantitative trait in the general population [15], and with the reversible changes associated with pregnancy and exercise [9,10]. Two further observations reinforce this position. First, the reported prevalence depends heavily on the imaging modality and the population: a systematic review and meta-analysis in adults found a prevalence of 14.8% by CMR against 1.3% by echocardiography, reaching 27.3% among athletes assessed by CMR [75]. Second, the reproducibility of the morphological criteria themselves is limited; in a study applying prespecified criteria, intra-institutional agreement reached a kappa of 0.79, whereas inter-institutional agreement was 0.63 to 0.67, judgements disagreed in 35% of cases, and 11% remained undecided even after adjudication [76]. The two modalities are not interchangeable: echocardiography underestimates trabeculation in the apical and lateral segments, whereas CMR detects trabeculation that is frequently present in healthy individuals, so the same patient may or may not meet a criterion depending on the modality and the cardiac phase used. Meeting a morphological criterion such as the N/C ratio therefore cannot by itself determine whether the finding signifies a cardiomyopathy. Two analyses published since have a direct bearing on this question. The first argues that the diagnostic label itself should be retired because the morphological criteria do not identify a distinct disease [77]. The second reports that hypertrabeculation in dilated cardiomyopathy does not carry independent prognostic weight once ventricular function, genotype and myocardial substrate are accounted for [78]. Both bear on the present framework, and it is worth being precise about how. They show that the morphological finding does not act as a risk modifier: within a population already defined by an established cardiomyopathy phenotype, the extent of trabeculation adds nothing to prognosis. This is an argument against the risk axis rather than against the category axis, and it is one reason the two are separated here.

### 6.2. Limitations of Genetic Information Alone

First, there is incomplete penetrance. Many carriers of DCM-associated P/LP variants in the general population do not manifest overt cardiomyopathy [79], and in HCM the manifestation among sarcomere variant carriers is age-dependent [80]. In familial LVNC cohorts as well, the clinical phenotypes of P/LP variant carriers differ greatly within the same family [81]. The presence of a P/LP variant alone therefore cannot establish a diagnosis of an overt cardiomyopathy.

Second, there is marked variable expressivity and genetic overlap with other cardiomyopathies. The same *MYH7* variant is found in multiple phenotypic groups, including the DCM phenotype, biventricular involvement, and CHD-associated hypertrabeculation [53], and *TAZ* likewise may produce phenotypes ranging within a family from severe infantile cardiomyopathy to mild adult LVNC [54,55]. A systematic review also found it difficult to predict the clinical phenotype of LVNC unequivocally from genotype [49].

Third, the genetic background is not complete at the level of a single gene. Multiple P/LP variants may modify severity but have not been established as independent predictors of outcome [12,53,64], and in HCM it has been shown that polygenic risk from common variants and environmental factors modify phenotypic expression [82,83]. Similar modifying effects are postulated in LVNC, but quantitative data are lacking. Why the same variant is accompanied by LVNC morphology in some carriers and not in others is not fully resolved, but at least three factors are postulated: the developmental stage at which the variant acts, since the ventricular wall grows through differential growth of the two layers and an abnormality operating during this window is readily expressed as relative excess of trabeculae; differences in hemodynamic loading, since volume loading and high-output states amplify trabeculation; and modifier genes and polygenic background. Genotype is thus important information that modifies the probability of an etiology and the level of risk, but it is not information that determines the category by itself.

### 6.3. Clinical Phenotype Alone Is Also Insufficient

Clinical phenotype has comparable limitations. DCM, HCM, and RCM are cardiomyopathy phenotypes based primarily on morphology and function, whereas the arrhythmia-predominant phenotype is described in terms of electrophysiological features, biventricular involvement in terms of anatomical distribution, and CHD-associated hypertrabeculation in terms of coexisting structural abnormality—that is, along different axes. Moreover, the same gene spans multiple phenotypes [49,53], and within the same phenotype the contributions of genetic etiology, underlying cardiac disease, and hemodynamic loading differ. Phenotypic subgroups are therefore useful for describing the clinical picture but are insufficient as etiological disease entities.

### 6.4. The Age and Time Axis Cannot Be Ignored

The clinical meaning of LVNC also changes with age and over time. Sarcomere gene variants are found at a high rate in fetal-onset cases [21], and severe phenotypes and death or cardiac transplantation are more frequent in fetal- and infantile-onset cases [22,72]. The distribution of causative genes may differ by age, and the genetic architecture itself differs between pediatric and adult cardiomyopathy [48,53]. Excessive trabeculation associated with pregnancy may also regress postpartum [9]. Rather than regarding a morphological finding at a single time point as a fixed disease attribute, a life-course perspective encompassing timing of onset, relationship to loading, and change over time is therefore required.

### 6.5. Multiple Pathophysiologic Pathways Revealed by Integration

Organized from the standpoint of pathogenetic mechanism, the pathways leading to left ventricular hypertrabeculation can be broadly divided into at least three. The first comprises primary abnormalities of endocardial–myocardial signaling controlling ventricular wall patterning, of cell fate determination, of proliferation, maturation, and polarity, of ECM metabolism, and of vascular–metabolic coupling (e.g., *MIB1*, *TBX20*, *PRDM16*, *NKX2-5*) [24,31,34,36,39,41,44,65]. The second comprises pathways in which cardiomyocyte-intrinsic structural, contractile, RNA-metabolic, and metabolic abnormalities interfere with developmental and maturation programs (e.g., *MYH7*, *ACTN2*) [13,21,46,47]. The third is the pathway in which trabeculation is amplified in response to hemodynamic loading [9,10]. These are not mutually exclusive, and more than one may be superimposed in a single patient. Figure 2B presents the same organization at the level of the gene groups in which these routes are encountered clinically: the first route corresponds to the developmental gene group, the second is subdivided into the sarcomeric, metabolic and mitochondrial, ion channel, nuclear envelope, cytoskeletal and RNA-binding groups, and the third is the load-dependent route, which acts postnatally and is largely reversible. The routes act in parallel rather than in sequence.

These three mechanistic pathways cannot, however, be mapped one to one onto three clinical categories. Longitudinal human developmental data are lacking, and many molecular mechanisms are based on the mouse and zebrafish. In addition, many of the animal models carrying these molecular mechanisms exhibit embryonic lethality, resulting in a substantial divergence from the human clinical phenotype [28]. Furthermore, conditions showing insufficient compact layer growth, as in the NAE1–Hippo–YAP and Rac1 models, and conditions showing ECM persistence with sustained cardiomyocyte proliferation, as in the CHD4 model, may both converge on excessive trabeculation [39,41,43]. The essence of the pathophysiology therefore lies not in the total amount of proliferation but in which region, at which stage, and which cellular program deviates. What is required in clinical practice is thus not the mechanical assignment of a category from a mechanism but an integrated judgment of the pathophysiologic context in which excessive trabeculation exists.

## 7. Three Clinical Pathophysiologic Contexts Derived from an Integrated Understanding

When normal development, pathogenetic mechanisms, genetics, clinical phenotypes, and the time axis are examined in sequence, it is difficult to treat left ventricular hypertrabeculation as a single disease entity. When organized from the perspectives of (i) the presence or absence of overt cardiomyopathy, (ii) the presence or absence of another underlying cardiac disease or pathological hemodynamic loading that could reasonably explain the excessive trabeculation, and (iii) the clinical course over time, cases can conceptually be understood as three clinical pathophysiologic contexts. These are referred to below, for convenience, as Categories 1–3 [3,4,5,84].

### 7.1. Purpose of the Framework and Its Three Axes

The purpose of this framework is not to rearrange existing classifications. It is to isolate a candidate disease entity by separating, from the heterogeneous population that shares this morphology, both the group in which excessive trabeculation is related to another cardiac disease or to pathological hemodynamic loading (Category 2) and the group that lacks cardiomyopathy-level functional abnormality (Category 3); the remainder is delimited as Category 1. This is the construction by which contemporary definitions of hypertrophic and dilated cardiomyopathy are themselves built, in which the phenotype is required not to be solely explained by abnormal loading conditions or by another disease [4].

The framework comprises three axes. Axis 1 is the category that concerns standing as a disease entity and takes the values Category 1, 2 or 3. Axis 2 is the clinical phenotypic group, a descriptive account of the coexisting morphological and functional features, comprising the seven groups of Section 5, which may overlap with one another. Axis 3 is the set of risk modifiers, which stratify risk and the intensity of follow-up within a category. The category is a judgement about standing as a disease entity and does not express magnitude of risk; risk is stratified within a category by Axis 3. This is the same construction as in hypertrophic cardiomyopathy, where diagnosis is one axis and sudden-death risk is stratified on a separate axis by modifiers such as family history, syncope and late gadolinium enhancement.

What the framework adds: Category 1 is defined by overt cardiomyopathy-level dysfunction or arrhythmia that is not otherwise explained and therefore identifies cardiomyopathy by definition; it does not by itself establish that excessive trabeculation has etiological significance. Whether the trabeculation contributes anything beyond delimiting the population is the question the framework is intended to make answerable. Its incremental value over classification by phenotype, genotype, function and myocardial substrate alone is threefold. First, the attribution judgement is at present made implicitly whenever a patient with trabeculation and ventricular dysfunction is labelled noncompaction cardiomyopathy; because it is not recorded, published cohorts mix all three Categories, which is one reason reported prevalence and outcome estimates differ by an order of magnitude, and making the judgement explicit is a precondition for assembling comparable cohorts. Second, separating standing as a disease entity from magnitude of risk prevents a morphological finding from determining anticoagulation, activity restriction or the intensity of family screening in Categories 2 and 3. Third, the framework is falsifiable: it predicts that outcomes in Category 2 track the underlying disease rather than the extent of trabeculation, that outcomes in Category 3 approach those of the general population, and that patients assigned to Category 1 and to Category 2 differ in natural history, genetic architecture and response to therapy once ventricular function and myocardial substrate are accounted for, since it is the attribution judgement, and not the extent of trabeculation, that distinguishes them. Whether the framework yields diagnostic, prognostic or therapeutic gain has not been demonstrated, and until it has, Category 1 is to be read as a candidate entity delimited for testing rather than as an established one.

Three points should be stated plainly at the outset. First, the attribution judgement on which the framework rests—whether the findings are explained by another disease or by loading—is the same construction as that already embedded in the ESC 2023 definitions of hypertrophic and dilated cardiomyopathy, in which the myocardial abnormality is required not to be explained by loading conditions alone; the presence of a judgmental element therefore does not by itself preclude standing as a disease entity. Second, the interobserver reproducibility of this framework has not been tested, and no numerical expectation can be offered; the reproducibility of the existing morphological criteria is itself limited [76], so this is a limitation of the field rather than of the framework alone. Third, and most importantly, it is not claimed here that Category 1 has been established as a disease entity. Establishing it would require (1) operationalization of the diagnostic requirements together with testing of interobserver reproducibility, (2) description of outcomes in a prospective cohort restricted to Category 1, (3) comparison of outcomes with Categories 2 and 3, and (4) international external validation.

Operational definition: In this review, an overt cardiomyopathy-level functional abnormality denotes any of the following: (1) Systolic dysfunction—a left ventricular ejection fraction below 50%, which is an adult threshold; in children, a value below the age- and body-size–specific normal range. The normative dataset, the parameter to be used (ejection fraction, fractional shortening or global longitudinal strain) and the corresponding Z-score threshold are not established in children, and defining them is a prerequisite for applying this framework to pediatric patients. (2) Diastolic dysfunction demonstrated by standard indices, such as a restrictive filling pattern. (3) Clinically important arrhythmia—sustained ventricular tachycardia, ventricular fibrillation, resuscitated cardiac arrest, arrhythmia accompanied by syncope, advanced atrioventricular block, or sinus node dysfunction requiring pacing. These thresholds are offered as an operational proposal and require validation.

### 7.2. The Three Clinical Pathophysiologic Contexts

**Figure 3:**
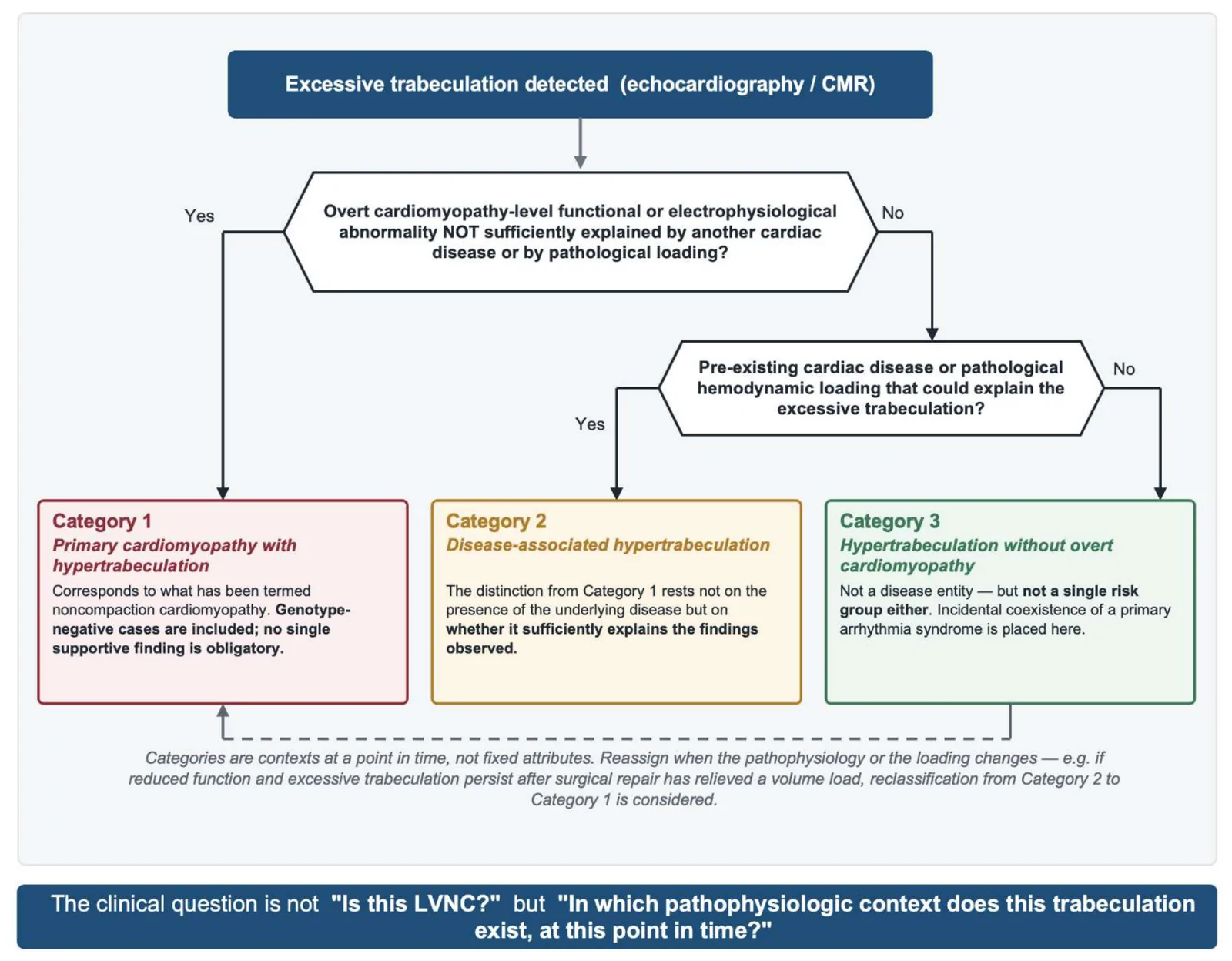
Assignment of the clinical pathophysiologic context of excessive left ventricular trabeculation. Assignment proceeds from two judgements: first, whether an overt cardiomyopathy-level functional or electrophysiological abnormality is present that is not sufficiently explained by another cardiac disease or by pathological hemodynamic loading; and second, whether a pre-existing cardiac disease or pathological hemodynamic loading capable of explaining the findings is present. Physiological loading, such as pregnancy or athletic training, is not treated as pathological loading at this step. The operational definitions of these judgements, the three resulting Categories, the risk modifiers within Category 3 and the recommended evaluation for each phenotypic group are given in Section 7.1 and Section 7.2 and in Table 2 and are not repeated here. In the systolic-dysfunction node, the pediatric threshold is not yet defined (Section 7.1). It is not claimed that Category 1 has been established as a disease entity. CMR, cardiac magnetic resonance; LVNC, left ventricular noncompaction; P/LP, pathogenic or likely pathogenic.

The two judgements and the three resulting contexts are summarized in Figure 3. Category 1 (primary cardiomyopathy with hypertrabeculation), corresponding to what has been termed noncompaction cardiomyopathy: a pathophysiologic context in which, in addition to excessive trabeculation, there are overt myocardial functional or electrophysiological abnormalities at the level of a cardiomyopathy, and in which these abnormalities cannot be sufficiently explained by another underlying cardiac disease or by pathological hemodynamic loading alone. The abnormalities referred to here include systolic dysfunction, diastolic dysfunction, and clinically important arrhythmias judged to be pathophysiologically related to the myocardial phenotype. P/LP variants in developmental, sarcomeric, ion channel-related, or metabolic and mitochondrial genes may be identified, but genotype-negative cases are also included. Compact layer thinning, early onset, family history, late gadolinium enhancement, and variants of established etiological significance may support this context, but none is an obligatory criterion on its own.

Category 2 (disease-associated hypertrabeculation): a clinical pathophysiologic context in which excessive trabeculation coexists with a pre-existing cardiac disease or pathological hemodynamic loading whose pathophysiology could reasonably explain it. Rather than asserting a temporal causal relationship as “secondary LVNC”, this is understood as a state in which the underlying disease and the hypertrabeculation are pathophysiologically related. The clinical course and therapeutic strategy are in principle determined by the pathophysiology of the underlying disease.

The distinction between Categories 1 and 2 does not rest on the mere presence or absence of a pre-existing cardiac disease or of pathological loading but on whether the excessive trabeculation actually observed is sufficiently explained by them. A case may therefore fall into Category 1 even when a pre-existing cardiac disease is present, if the abnormality observed exceeds what the severity of that disease would explain. Where the coexisting disease is itself a dilated or hypertrophic phenotype, this judgement turns on the temporal sequence: excessive trabeculation that precedes the functional abnormality, or that is first documented together with it before the ventricle is remodeled, is not explained by the remodeling and indicates Category 1, whereas excessive trabeculation that appears after such a phenotype has been documented is explained by it and indicates Category 2, since dilatation with thinning of the compact layer and hypertrophy with increased prominence of the trabeculae both raise the noncompacted-to-compacted ratio on geometric grounds alone.

Category 3 (hypertrabeculation without overt cardiomyopathy): a clinical pathophysiologic context in which excessive trabeculation is present but no overt functional or electrophysiological abnormality at the level of a cardiomyopathy is currently identified, and in which there is no pre-existing cardiac disease or pathological hemodynamic loading capable of explaining the trabeculation. Cases in which an established primary arrhythmia syndrome coexists incidentally are also placed here, with the arrhythmia syndrome being managed separately as an independent comorbid disease (Section 7.4). Category 3 does not constitute a disease entity but neither is it a single risk group.

Risk modifiers within Category 3 are (1) a P/LP variant of established etiological significance; (2) a family history of cardiomyopathy or of premature sudden death; (3) a morphological abnormality such as compact layer thinning; (4) an electrocardiographic abnormality; and (5) the absence of a physiological trigger. On this basis, three situations are distinguished. In the physiological and reversible situation, an evident trigger is present, no modifier is identified, and the findings improve after the loading is relieved; overdiagnosis should be avoided, and cardiomyopathy-level follow-up is not required. In the isolated and indeterminate situation, neither a physiological trigger nor a modifier is evident; morphology, cardiac function and the electrocardiogram are re-evaluated at intervals without assigning a disease name. In the situation with risk modifiers, risk is assessed individually according to the modifier concerned, and shorter re-evaluation intervals are considered where compact layer thinning is present. Category 3 is therefore not synonymous with “physiological”; the need for and intensity of follow-up differ according to these modifiers. These recommendations are expert proposals derived from indirect evidence. The listed modifiers have not been validated as prognostic predictors in individuals with preserved cardiac function, and the absence of a physiological trigger is an attribution criterion rather than an established risk factor; it is used here only to indicate that a physiological explanation for the finding is not available.

### 7.3. Categories Are Not Fixed Attributes

Categories are contexts at a point in time and require reassignment as the pathophysiology or the loading conditions change. If, for example, excessive trabeculation and reduced cardiac function are observed under the volume loading of a ventricular septal defect and the case is assigned to Category 2, then persistence of the reduced function and of the excessive trabeculation after surgical repair has relieved the loading should prompt consideration of reclassification to Category 1; conversely, improvement of the findings after the loading is relieved supports the original assignment to Category 2. Similarly, a patient in Category 3 who develops a functional abnormality during follow-up should first be re-evaluated for an alternative disease or a loading condition that would account for it, and is reassigned to Category 1 only if none is identified. Reassignment changes the reasoning applied to a patient and the intensity of surveillance; it does not by itself alter treatment, which continues to follow the indications established for cardiac function, arrhythmia and heart failure.

### 7.4. Phenotypic Pattern and Pathophysiologic Context Are Not Synonymous

The phenotypic patterns presented in Section 5 (Axis 2) and Categories 1–3 (Axis 1) are concepts at different levels, and they do not correspond on a one-to-one basis. Throughout this correspondence, the second judgement asks whether the coexisting disease or loading explains the excessive trabeculation, not whether it explains the functional abnormality, which has already been settled at the first judgement. The DCM, HCM, and RCM phenotypes are therefore treated by the same rule (Section 7.2): Category 1 where the trabeculation precedes or accompanies the functional abnormality or exceeds what the remodeling would explain, Category 2 where it appears after the phenotype has been documented, and not yet assigned where the temporal sequence cannot be determined. Because the same patient may satisfy more than one phenotypic group, the category cannot be determined unequivocally from the phenotypic group, and even within the same phenotype the final pathophysiologic context may differ if the underlying disease, functional abnormality, genetic background, or timing of onset differs (Table 2) [12,53,68,69,70].

The arrhythmia-predominant phenotype requires this distinction most explicitly. Where the arrhythmic and the structural phenotype arise from the same pathophysiology—an electromechanical overlap phenotype—the case belongs to Category 1. Findings that support such relatedness include an association of the gene or variant concerned with the structural phenotype, as for pore or transmembrane variants of *HCN4* and the *RYR2* exon 3 deletion [13,50,51,52]; co-segregation of both phenotypes within the family; and structural findings not readily explained by incidental coexistence. These are supporting findings rather than threshold criteria. Where, by contrast, the diagnosis of a primary arrhythmia syndrome is established, the variant concerned shows no association with a structural phenotype, the excessive trabeculation is isolated with a preserved compact layer, cardiac function is normal and non-progressive, and carrier relatives show no structural phenotype, incidental coexistence should be judged, and the case is not assigned to Category 1. Such a case does not belong to Category 2 either, since the excessive trabeculation is not explained by the pathophysiology of the primary arrhythmia syndrome; it is placed in Category 3, and the arrhythmia syndrome is managed separately according to the guidance for that condition. Here, the genetic finding is used not to determine the category by itself but as one basis for judging whether two coexisting phenotypes arise from a common pathophysiology. Where neither judgement can be reached, Category 1 is not assigned; the case is followed as Category 3 and re-evaluated if structural findings progress or cardiac function declines.

### 7.5. Clinical Implications: What Should Be Integrated in the Evaluation

The purpose of this framework is not to determine treatment automatically from a category label (Figure 3) but to return the morphological finding to its clinical pathophysiologic context for evaluation. Age at onset, change over time, and family history are important; fetal or infantile onset and familial clustering of cardiomyopathy suggest primary cardiomyopathy [21]. Conversely, regression of trabeculation after relief of loading supports a reversible background [9].

Genetic information should be interpreted in integration with morphology, cardiac function, clinical phenotype, and family history. Death or cardiac transplantation and worsening heart failure have been reported more frequently in patients carrying multiple P/LP variants and in specific gene groups, but their independent predictive value is not uniform [12,53,64]. Accordingly, a case should not be assigned to Category 1 on the basis of the presence of a P/LP variant alone, nor should a primary cardiomyopathy be excluded on the basis of a negative genetic test alone. In children without an evident physiological trigger, a three-generation family history should be taken as a minimum—covering cardiomyopathy, heart failure, premature sudden death, unexplained fatal accidents, and use of a pacemaker or implantable cardioverter-defibrillator—together with screening of first-degree relatives by echocardiography and electrocardiography. Genetic testing should be based on a panel covering cardiomyopathy-associated genes, with the indication, the supporting arrangements and the disclosure of results following the relevant guidance [67]. Because a negative genetic test does not exclude genetic predisposition, the follow-up policy for such cases should be determined not by the genetic findings alone but by morphological abnormality, family history, electrocardiographic findings and change over time.

Morphologically, evaluation should extend beyond the N/C ratio to the absolute and relative thickness of the compact layer, ventricular volume, wall thickness, cardiac function, and tissue characteristics such as late gadolinium enhancement and T1/ECV. In pediatric cohorts, reduced compact layer thickness was associated with death or cardiac transplantation [72], and in adults, regional thinning of the compact layer was associated with major adverse cardiac events [61]. An association between compact layer thinning and lower systolic function has also been reported [62]. Age- and size-specific normative values for compact layer thickness are nevertheless not established in children, and compact layer thickness, although promising as an outcome indicator, is not a validated criterion for category assignment. In this framework it is positioned not as a branching condition for the category but as a risk modifier within Category 3 and as a finding supporting pathophysiologic relatedness.

In Category 1, the individual risks of heart failure, diastolic dysfunction, arrhythmia, and thromboembolism should be assessed and managed in accordance with existing guidelines for cardiomyopathy [4]. Indications for an implantable cardioverter-defibrillator, cardiac resynchronization therapy, or evaluation for cardiac transplantation are determined by the general criteria applied to cardiomyopathy—cardiac function, symptoms, arrhythmia, and a family history of syncope or sudden death. Genetic information is useful for refining the diagnosis, for family screening, and for setting the follow-up policy, but direct evidence supporting genotype-based determination of device indications in LVNC is at present limited; where a case is assigned to Category 1, device indications continue to follow the established criteria for the arrhythmia syndrome concerned. In Category 2, evaluation and treatment based on the pathophysiology of the underlying disease are central, and the excessive trabeculation is recorded as an accompanying finding. In Category 3, management follows the three situations set out in Section 7.2.

Thromboembolism is less frequent in children than in adults with this morphology: in a systematic review and meta-analysis of 726 pediatric and 3862 adult patients, the prevalence was 2.6% and 6.2% and the annual incidence 1.4% and 2.9%, respectively, and in the pediatric subset a left ventricular ejection fraction below 40% was the only factor independently associated with thromboembolism, although the estimate was imprecise (odds ratio 9.47, 95% confidence interval 1.35–188.2) [85]. Hypertrabeculation itself was not significantly associated with embolic events among 1160 adults with dilated cardiomyopathy assessed by cardiac magnetic resonance (hazard ratio 1.5, 95% confidence interval 0.75–3.00), an estimate too imprecise to exclude a clinically relevant increase, whereas atrial fibrillation and reduced ejection fraction were [78]. Anticoagulation therefore follows the established indications—atrial fibrillation, intracardiac thrombus, a previous embolic event, or another standing indication—rather than the morphological finding; reduced ejection fraction in sinus rhythm is not by itself an indication in adults under current heart failure guidance. In children, no threshold has been validated, and although severe systolic dysfunction leads some centers to consider anticoagulation, this is institutional practice rather than guideline recommendation; excessive trabeculation is not an independent indication in any Category.

### 7.6. Limitations of This Conceptual Framework and Future Validation

This framework has several limitations. First, longitudinal human data tracking the course from normal development to the establishment of pathological hypertrabeculation are scarce, and many molecular mechanisms depend on animal models. Second, developmental abnormality, cardiomyocyte-intrinsic abnormality, and hemodynamic loading overlap with one another, and an individual case cannot always be reduced to a single etiology. Third, because Category 3 encompasses both physiological change and preclinical cardiomyopathy, valid thresholds for the risk modifiers and for follow-up intervals remain undetermined. Fourth, the operational definition proposed in Section 7.1 has not been validated, and the interobserver reproducibility of the attribution judgement has not been tested. Fifth, these three pathophysiologic contexts are not an externally validated diagnostic classification or risk score. In particular, because Category 1 by definition includes overt functional and electrophysiological abnormalities, simply asserting that Category 1 is associated with death or cardiac transplantation would create definition–outcome circularity. Testing future performance will require evaluation, in prospective multicenter cohorts, of independence and incremental value relative to known factors such as age, LVEF, late gadolinium enhancement, genotype, and underlying disease, together with the four requirements for establishing a disease entity set out in Section 7.1. The pediatric threshold for systolic dysfunction in the operational definition also remains to be defined (Section 7.1). The framework is refutable, and the terms should be stated exactly. If cohorts assembled by the attribution judgement do not differ from cohorts assembled without it—in natural history, in genetic architecture, or in response to therapy, once ventricular function and myocardial substrate are accounted for—then the judgement carries no information, and Category 1 has no independent standing.

## 8. Conclusions

Excessive left ventricular trabeculation, which has been grouped together as LVNC, is more consistent with current evidence when understood not as a single developmental defect or a single inherited cardiomyopathy but as a common morphological end point on which multiple developmental, genetic, cardiomyocyte-intrinsic, and hemodynamic pathways converge. Research on normal ventricular wall formation has updated the classical “arrest of compaction” model to the concept of differential growth, and genetics has shown that LVNC has a diverse background extending from developmental genes to sarcomeric, ion channel, and metabolic and mitochondrial genes. Clinically, as well, the DCM, HCM and RCM phenotypes, arrhythmia predominance, biventricular involvement, CHD association, and preserved function overlap with one another, and the entity cannot be organized along any single axis of morphology, genotype, or phenotype.

When these findings are integrated in sequence, it is clinically more intelligible to organize left ventricular hypertrabeculation into three contexts: existing as a primary cardiomyopathy with hypertrabeculation, existing in relation to another cardiac disease or pathological loading, and existing without overt cardiomyopathy. The purpose of this organization is to isolate, from a heterogeneous population, a candidate disease entity that can be examined, while the category itself is a judgement about standing as a disease entity, and risk is stratified within a category by separate modifiers. These three are not a fixed or mutually exclusive etiological classification but clinical pathophysiologic contexts that may change with the patient’s age, the passage of time, underlying disease, cardiac function, and genetic background. Their value therefore lies not in adding a new “LVNC classification” but in ensuring that, when excessive trabeculation is encountered, the diagnosis is not completed on the basis of morphology alone, and the underlying pathophysiology is re-examined in an integrated manner. It is not claimed that Category 1 has been established as a disease entity; operationalization of the diagnostic requirements with testing of interobserver reproducibility, description of outcomes in a cohort restricted to Category 1, comparison with Categories 2 and 3, and international external validation are required, particularly in longitudinal cohorts spanning childhood to adulthood.

## Data Availability

All data supporting the findings of this study are available within the paper.
